# Targeting epigenetics in sarcomas through EZH2 inhibition

**DOI:** 10.1186/s13045-020-00868-4

**Published:** 2020-04-07

**Authors:** Antoine Italiano

**Affiliations:** 1 Institut Bergonié, Early Phase Trial and Sarcoma Units, 229 cours de l’Argonne, 33076 Bordeaux, CEDEX France; 2 INSERM U1218, Bordeaux, France; 3 University of Bordeaux, Bordeaux, France

## Abstract

Soft-tissue sarcomas represent a heterogeneous group of diseases with distinct genetic and clinical features accounting for up to 1% of cancer in adults and 15% of cancer in children. Epithelioid sarcoma is an extremely rare and aggressive tumor affecting young adults that is characterized by loss of INI1 expression. INI1 (SMARCB1, SNF5, BAF47) is a subunit of the SWI/SNF chromatin remodeling complex that opposes the enzymatic function of EZH2. When INI1 loses its regulatory function, EZH2 activity is de-regulated, allowing EZH2 to play a driving, oncogenic role. Tazemetostat, a specific EZH2 inhibitor, has just been approved for patients with advanced epithelioid sarcoma and represents a new therapeutic option in this devastating disease.

Over the past three decades, outcomes of therapy have improved considerably for a wide range of cancers. For example, the discovery of new chemotherapeutic agents and the optimization of multimodality therapies have improved the cure rate for pediatric cancers from less than 10% in the 1950s to over 80% today; similarly, the 5-year survival rate of testicular and breast cancer improved from 57% and 60% in the 1950s to over 96% and 90%, respectively, today. Unfortunately, little progress has been made in terms of survival for patients with soft-tissue sarcomas (STS).

STS represent a heterogeneous group of rare tumors including more than 70 different histological subtypes [1]. Five and 30% of sarcomas are diagnosed in patients less than 20 years old and more than 75 years old, respectively.

Many of the STS subtypes have probably specific mechanisms of oncogenesis and might, therefore, be especially sensitive to appropriate systemic treatments. The identification of new therapies for STS patients is of crucial importance. Indeed, 40 to 50% of patients with STS will develop metastatic disease. Once metastases are detected, the treatment is mainly based on palliative chemotherapy. Since its approval in 1974, doxorubicin remains nowadays the first-line standard of care and the median survival of patients in this setting ranges between 12 and 20 months [2]. Therefore, it is generally acknowledged that the benefits from chemotherapy in these diseases have reached a plateau and that new therapeutic strategies are urgently needed.

Despite recent insights into sarcoma genetics, a driver genetic aberration that can serve as a therapeutic target has been identified in only a minority of sarcomas. INI1 (SMARCB1/SNF5/BAF47) gene aberration represents one of them. INI1 is a potent tumor suppressor gene, a member of the SWI/SNF complex whose integrated functions control diverse cellular processes such as differentiation and proliferation [3]. Loss of INI1 function leads to elevated expression and recruitment of EZH2 to target genes that become trimethylated on H3K27 and repressed [3], which results in the upregulation of several oncogenic signaling pathways, including Sonic Hedgehog, Wnt/β-Catenin, and MYC [3]. INI1 loss was first identified in malignant rhabdoid tumors (MRTs) which are rare and aggressive cancers that principally occur in childhood and can arise in various locations, mainly the kidney, brain, and soft tissues. MRTs harbor recurrent and specific biallelic-inactivating mutations or deletions of INI1 located in the 22q11.2 region [4]. Interestingly, apart from this specific alteration, MRTs have a remarkably low rate of mutations and no genomic instability [5], suggesting a potential oncogenic driver role of INI1 loss in MRT tumorigenesis. INI1 loss has also been found with high frequency (50 to 80%) in epithelioid sarcoma (ES) or other sarcomas with epithelioid features such as malignant peripheral nerve sheath tumors (MPNST) [6, 7].

Preclinical data showed that EZH2 inhibition leads to specific repression of cellular H3K27 methylation and induces apoptotic death of INI1-negative MRT cells [8, 9]. These findings suggest a synthetic–lethal interaction between INI1 and EZH2 and consequently offer a promising therapeutic approach in this disease. Tazemetostat (EPZ-6438) is a potent and highly selective EZH2 inhibitor [9, 10] that has shown activity in INI1-negative MRT cells, both in culture and in xenograft experiments in vivo. In 2013, a phase 1 trial was initiated to evaluate the safety and toxicity profile of daily oral administration of tazemetostat in patients with metastatic or locally advanced solid tumors or non-Hodgkin lymphoma (NHL) (NCT01897571) [11]. In June 2014, we enrolled in this study the first patient with INI1-negative solid tumor. This patient who suffered from a relapsed MRT displayed a complete response which lasted for more than 4 years. This event prompted the enrolment of additional patients with these genetic lesions to more fully evaluate the activity and safety of the drug in this population. We observed clinical activity consisting of objective responses (complete responses and partial responses) or prolonged stable disease (6.4 to > 20 months), which has exceeded a duration of 2 years in five (38%) of 13 patients with INI1-negative or SMARCA4-negative solid tumors [11]. Interestingly, none of the patients with tumors bearing wild-type expression of INI1 or SMARCA4 proteins had an objective response. Tazemetostat was well tolerated, with most treatment-related adverse events being grades 1 or 2 (asthenia, anorexia, thrombocytopenia, nausea, and dyspnea). These encouraging preliminary results led to the design of a basket phase 2 study investigating tazemetostat in INI1-negative tumors (NCT02601950). With only 2 objective responses among 31 patients, stage 2 futility was not passed in the rhabdoid tumor cohort [12]. The results obtained in the epithelioid sarcoma cohort are more promising and potentially practice-changing [13]. Epithelioid sarcoma is an extremely rare sarcoma that affects young adults [14]. Surgical resection is the cornerstone of treatment for localized disease. In the advanced setting, existing cytotoxic drugs (including doxorubicin) are associated with modest efficacy. Sixty-two patients (24 in the first-line setting and 38 who already received systemic therapy) were enrolled in the tazemetostat study [15]. At data cutoff, the overall response rate was 15% [9/62, 95% CI, 6.9–25.8] (25% for patients in the first-line setting and 8% for those who had prior systemic therapies). At a median follow-up of 59.9 weeks, the median duration of response was not reached. The overall disease control rate (partial response or stable disease ≥ 32 weeks) was 26% (95% CI 15.5–38.5). The median progression-free survival was 23.7 weeks (95% CI, 14.7–25.7), and the median overall survival was 82.4 weeks (95% CI, 47.4–NE). As observed in the phase I study, the safety profile of tazemetostat was good and compared favorably with that of commonly used cytotoxic drugs such as doxorubicin and gemcitabine. Altogether, these results showed that tazemetostat can be associated with substantial clinical benefit in a subset of patients with advanced epithelioid sarcoma. Based on these data, the United States Food and Drug Administration (USFDA) granted accelerated approval to tazemetostat for the treatment of adults and pediatric patients aged 16 years and older with metastatic or locally advanced epithelioid sarcoma not eligible for complete resection in January 2020. Therefore, tazemetostat (TazverikTM) is the first epigenetic drug approved for the treatment of patients with solid tumors. However, several questions regarding the role of tazemetostat in the treatment of patients with epithelioid sarcoma will require further investigations.

In comparison with the rhabdoid tumor (the other major group of INI1-negative cancers), epithelioid sarcoma shows a relatively high level of genomic aberrations [16]. Although all cases are characterized by loss of INI1 expression, more than 30% of them have no genetic aberration of *INI1* [16]. The mechanism of INI1 protein loss in this *INI1*-wild type epithelioid sarcoma is not fully understood and may involve epigenetic events such as methylation. Preliminary data have suggested that DNA methylation profile may correlate with the outcome of patients on tazemetostat but require further confirmation before this can be considered as a potential predictive biomarker [16]. Finally, the excellent safety profile of tazemetostat may allow a potential combination with other agents. A combination with cytotoxic drugs may represent a promising approach as suggested by pre-clinical data showing synergy between tazemetostat and doxorubicin [17]. A phase Ib investigating the safety of tazemetostat in combination with doxorubicin is ongoing (NCT04204941), and a phase III that will compare doxorubicin versus doxorubicin combined with tazemetostat in the front-line setting for epithelioid sarcoma treatment is planned to open accrual in 2020. In addition to cytotoxic drugs, immune checkpoint inhibitors (ICI) can represent another interesting class of agents to combine with tazemetostat. We have reported a strong induction of CD8 T cells in a patient with epithelioid sarcoma treated with tazemetostat [11]. This immune infiltrate was neither present at baseline nor in a later specimen collected at disease progression. Several studies have shown the role of EZH2 in immunomodulation [18]. Together with the recent demonstration that EZH2 inhibition enhances ICI efficacy in pre-clinical models of melanoma and other solid tumors [19, 20], these findings pave the way for clinical trials combining ICI with tazemetostat in epithelioid sarcoma and other INI1-negative solid tumors.

## Data Availability

Data sharing is not applicable to this article as no datasets were generated or analyzed during the current study.
